# Cut‐Off Values Able to Identify Migraine Patients With Increased Pressure‐Pain Sensitivity Independent of the Migraine Cycle Through a Single Assessment: A Secondary Analysis of a Multicentre, Cross‐Sectional, Observational Study

**DOI:** 10.1002/ejp.4787

**Published:** 2025-01-21

**Authors:** Matteo Castaldo, Lars Arendt‐Nielsen, Marta Ponzano, Francesca Bovis, Paola Torelli, Cinzia Finocchi, Stefano Di Antonio

**Affiliations:** ^1^ Department of Health Science and Technology, Center for Pain and Neuroplasticity (CNAP), SMI, School of Medicine Aalborg University Aalborg Denmark; ^2^ Department of Medicine and Surgery, Clinical Psychology, Clinical Psychophysiology and Clinical Neuropsychology Labs University of Parma Parma Italy; ^3^ Department of Gastroenterology & Hepatology, Mech‐Sense, Clinical Institute Aalborg University Hospital Aalborg Denmark; ^4^ Steno Diabetes Center North Denmark, Clinical Institute Aalborg University Hospital Aalborg Denmark; ^5^ Section of Biostatistics, Department of Health Sciences (DISSAL) University of Genova Genova Italy; ^6^ Department of Medicine and Surgery, Headache Centre University of Parma Parma Italy; ^7^ Ospedale San Paolo, ASL 2 Savonese Savona Italy; ^8^ Department of Neuroscience, Rehabilitation, Ophthalmology, Genetics and Maternal Child Health University of Genova Genova Italy

**Keywords:** migraine, pressure pain threshold, sensitisation, sensory profiling

## Abstract

**Aim:**

Identify values that could predict the presence of increased pressure‐pain sensitivity independent of the migraine cycle through a single assessment.

**Methods:**

This was a secondary analysis of a previous study in which 198 episodic and chronic migraine patients were assessed during all phases of the migraine cycle. Pressure pain threshold (PPT) was assessed over the temporalis, cervical spine, hand, and leg. Migraine patients were divided into two sub‐groups: patients with increased pressure‐pain sensitivity (IPS) and with No IPS (No‐IPS). A Chi‐squared Automatic Interaction Detection decision tree analysis was used to identify predictors to be included in the IPS or NoIPS group. To assess the internal validity of the model, a tenfold cross‐validation was applied.

**Results:**

161 (81%) patients were included in the IPS group, while 37 (19%) in the NoIPS group. Migraine patients with: (1) Temporalis PPT ≤ 130 kPa; (2) Temporalis PPT > 130 kPa and ≤ 197.5 kPa and hand PPT ≤ 347.33 kPa; (3) Temporalis PPT > 197.5 kPa and hand PPT ≤ 315 kPa; were correctly included in the IPS group with a sensitivity of 96%, a specificity of 81%, a positive predictive value of 96%, and a negative predictive value of 81%. The accuracy of the model and the cross‐validation analysis were respectively 93% and 92%.

**Conclusion:**

The high internal validity suggests that our model could precisely predict the presence of IPS independently by the phase in which the assessment occurred. Trigeminal and hand PPT cut‐off values could be used to identify patients with IPS.

## 1 Introduction

Migraine is a common neurovascular brain disorder characterised by a cyclic increase in sensitisation mechanisms (Di Antonio, Arendt‐Nielsen, et al. 2022; Di Antonio, Castaldo, et al. 2022). Recently, it has been shown that increased pain sensitivity detected through Quantitative Sensory Testing (QST) could be a predictor of the treatment response in migraine patients (Hong et al. 2023), outlining the possible importance of performing such quantitative assessment. In a clinical setting (e.g., migraine), where no control groups are available, reference values are needed to identify patients with increased pain sensitivity, and normative cut‐off values extrapolated from healthy subjects have been proposed (Andersen et al. 2015). However, due to the cyclic nature of migraine, QST results vary across the migraine cycle (Peng and May 2018), reducing the clinical utility of using single normative cut‐off values from healthy subjects. Even if a migraine patient could be above a “cut‐off” threshold during the assessment, he/she may be below that same threshold the following days, in which he/she will be in a different phase of the migraine cycle (or vice versa).

To overcome this limit, we assessed migraine patients across the 4 phases of the migraine cycle and identified those with increased pressure‐pain sensitivity in each phase (pre‐ictal, ictal, peri‐ictal, inter‐ictal). Episodic and chronic migraine patients were included and divided into two cohorts according to the phase in which the examination occurs: 100 ictal/peri‐ictal patients (ictal: headache attack during the assessment; preictal: headache attack occurred in the 24 h after the assessment; postictal: headache attack occurred in the 24 h before the assessment); 98 interictal (no headache during the assessment or in the 24 h before/after the assessment). In each cohort, patients were sub‐grouped according to clinical and psychophysical variables into distinct clusters. The cluster analysis identified in each cohort a group with no increased pressure‐pain sensitivity (NoIPS) (18%–19% of patients), while the remaining 81%–82% of patients had increased pressure‐pain sensitivity (IPS) (Di Antonio et al. 2023). The IPS groups of both cohorts had reduced Pressure Pain Threshold (PPT) over all tested areas (temporalis, cervical spine, hand, and leg) compared to NoIPS and a group of healthy subjects matched for age, sex, and body mass index (*N* = 56) (Di Antonio et al. 2024). On the other hand, the NoIPS groups of both cohorts had either no significant difference or increased PPT compared with healthy subjects (Di Antonio et al. 2024). Finally, for each cohort, cut‐off values to identify migraine patients with IPS were identified (Di Antonio et al. 2023).

However, migraine patients were treated as two district cohorts according to the phases in which they were assessed, and cut‐off values to identify patients with IPS varied according to the phase in which the examination occurred (Di Antonio et al. 2023). This undermines the utility of these cut‐off values, as the phase in which the assessment took place could only be determined retroactively, after the development of the following headache attack. Thus, in this secondary analysis of the previous study (Di Antonio et al. 2023), we treated the two cohorts of migraine patients as one group, and we aimed to identify variables predictive of the presence of IPS independent of the interictal and ictal/peri‐ictal phases of migraine.

## 2 Method

This multicentre, cross‐sectional, observational study was conducted at the Parma and Genova Headache Center and approved by the Ligurian (244/2018) and “Area Vasta Emilia‐Nord” (18305/2019) regional ethics committee. All subjects signed an informed consent form and were assessed between April 2019 and February 2022.

This is a secondary analysis of a previous study (Di Antonio et al. 2023) in which a total of 198 episodic and chronic migraine patients were assessed during all phases of the migraine cycle. According to the phase in which the examination occurred, patients were categorised as being in the ictal/peri‐ictal phase (if the headache was present during the evaluation or occurred 24 h before or after the assessment) or in the interictal phase (no headache was present during the evaluation or in the 24 h before/after the assessment), as reported elsewhere (Di Antonio et al. 2023, 2024). The migraine diagnosis was performed according to ICHD‐3 criteria (Olesen 2018). Inclusion and exclusion criteria were explained in detail elsewhere (Di Antonio et al. 2023, 2024).

Migraine patients in each phase were then divided into two sub‐groups according to the results of a cluster analysis conducted in the primary analysis of these data. Briefly, clinical and psychophysical variables were assessed in each patient (headache frequency, disability, active range of motion of the cervical spine, and PPT). PPT was assessed using a hand‐held algometry (Somedic AB, Sweden, probe area 1 cm^2^, 30 kPa/s force increase) over the trigeminal area (anterior, middle, and posterior column of temporalis muscles), cervical spine (left and right C1, C2, C4, and C6 articular pillars), second metacarpophalangeal joint of the dominant hand, and tibialis anterior muscle of the dominant leg. These measurement points were chosen as they have been previously used to assess widespread hyperalgesia (Fernández‐de‐las‐Peñas et al. 2022). Trigeminal PPT was assessed over the symptomatic side in patients with unilateral migraine and over the dominant side in patients with side/shift or bilateral migraine.

Using these variables, a cluster analysis was performed with the K‐means algorithm, without making any a priori assumptions about the expected number of clusters. As a result, two subgroups were found, one showing reduced PPT values (IPS group) and one showing increased PPT values (NoIPS group) (Di Antonio et al. 2023). As in the primary analysis the clustering was performed controlling for the phase of the migraine cycle in which the assessment occurs, each patient was assigned to one group considering the phase in which the evaluation took place. The IPS group exhibited increased pressure‐pain sensitivity, while the NoIPS group exhibited decreased pressure‐pain sensitivity in all phases of the migraine cycle (Di Antonio et al. 2023). The complete procedure of the assessment and analysis was explained in detail elsewhere (Di Antonio et al. 2023).

After patients were assigned to the IPS or the NoIPS group, differences in sex, age, body mass index (BMI), and PPT were assessed across IPS migraine, NoIPS migraine, and a group of healthy controls without migraine or any other primary headache type with no family history of migraine or other primary headaches. As age and BMI were not normally distributed, differences in these variables were investigated with the Kruskal Wallis test. Differences in sex were investigated with the Chi‐square test. The Mann–Whitney test and the Chi‐square test (2 × 2 contingency table) were used to run post hoc analyses respectively for the Kruskal Wallis test and Chi‐square, using a Bonferroni corrected *p*‐value. PPT differences were investigated by performing ANCOVA (correcting for age, sex, and BMI) using log‐transforming PPT values. Bonferroni‐adjusted post hoc analysis was performed to make single‐groups comparisons.

Finally, a Chi‐squared Automatic Interaction Detection (CHAD) decision tree analysis was used to identify clinical predictors to be included in the NoIPS or IPS group. CHAD algorithm was chosen as it has already been used efficiently for prediction in a smaller sample of migraine patients (Pan et al. 2022). To shorten the assessment procedure and make it easily performed in a clinical scenario, only two independent PPT variables were used to predict the inclusion in the NoIPS or IPS group. These variables were PPT over the anterior column of the temporalis muscle (symptomatic side in patients with unilateral migraine; dominant side in patients with side/shift or bilateral migraine) and the second metacarpophalangeal joint of the dominant hand. Adjustments were as follows: maximum of 3 levels; minimum number of cases for parent nodes, *n* = 11; child nodes, *n* = 3. The Likelihood ratio was chosen as a statistic and the significance level for splitting nodes was set to *α* = 0.05 applying classical Bonferroni correction to avoid *α* error accumulation. Then, to assess the internal validity of the model, a tenfold cross‐validation was applied. The sample was divided into 10 equal parts, and 10 new decision trees were repeatedly refitted with 9/10 of the data. The model error for cross‐validation was calculated based on the average value of the 10 results and was compared to those of the main analysis. The statistical analyses were performed using the SPSS software (version 24).

## 3 Results

**FIGURE 1:**
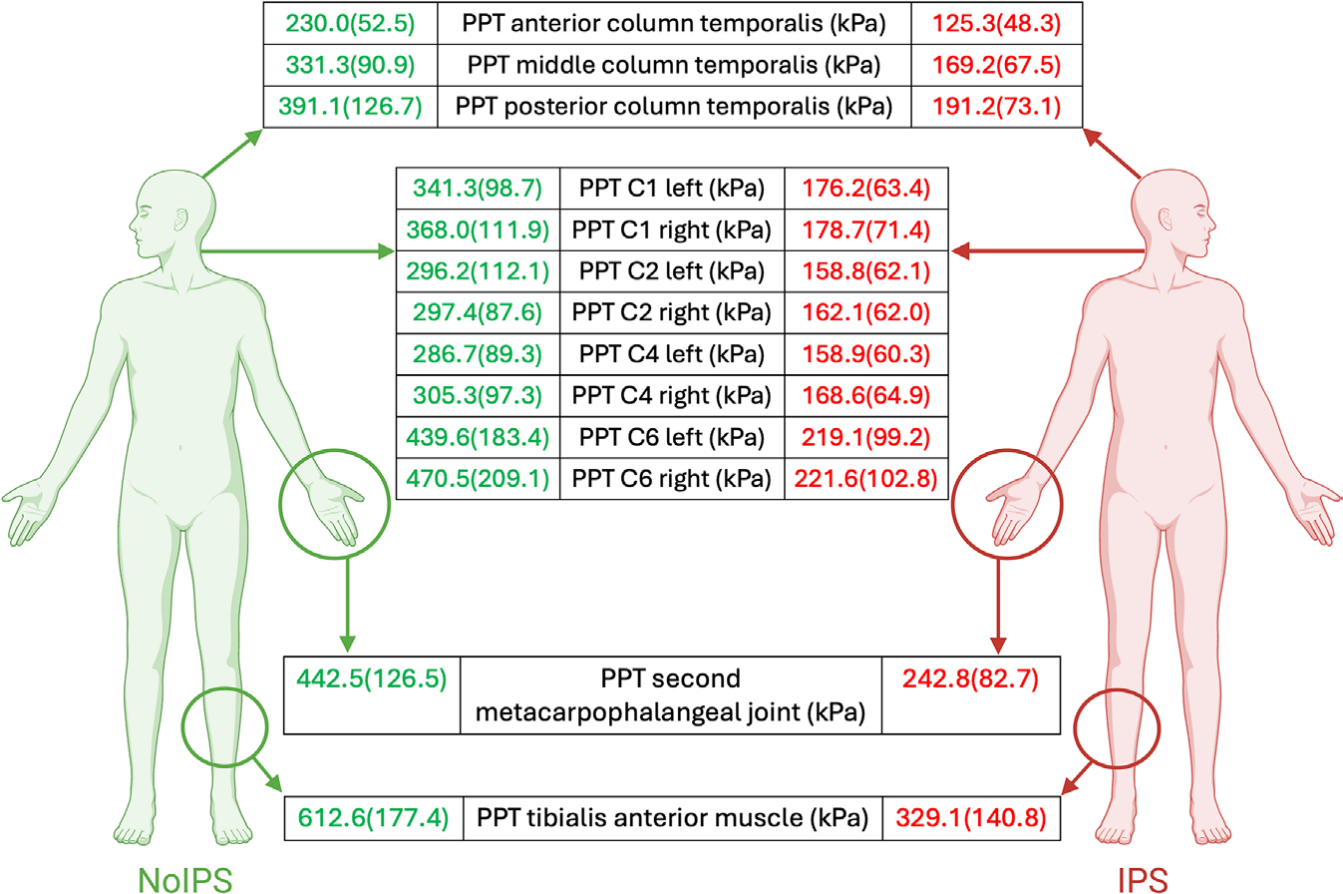
Sensory profile of migraine patients with no increased pressure‐pain sensitivity and with increased pressure‐pain sensitivity. PPT data were reported as mean and standard deviation. IPS, increased pressure‐pain sensitivity; kPa, kilopascal; NoIPS, no increased pressure‐pain sensitivity; PPT, pressure pain threshold. Created with BioRender.com.

**FIGURE 2:**
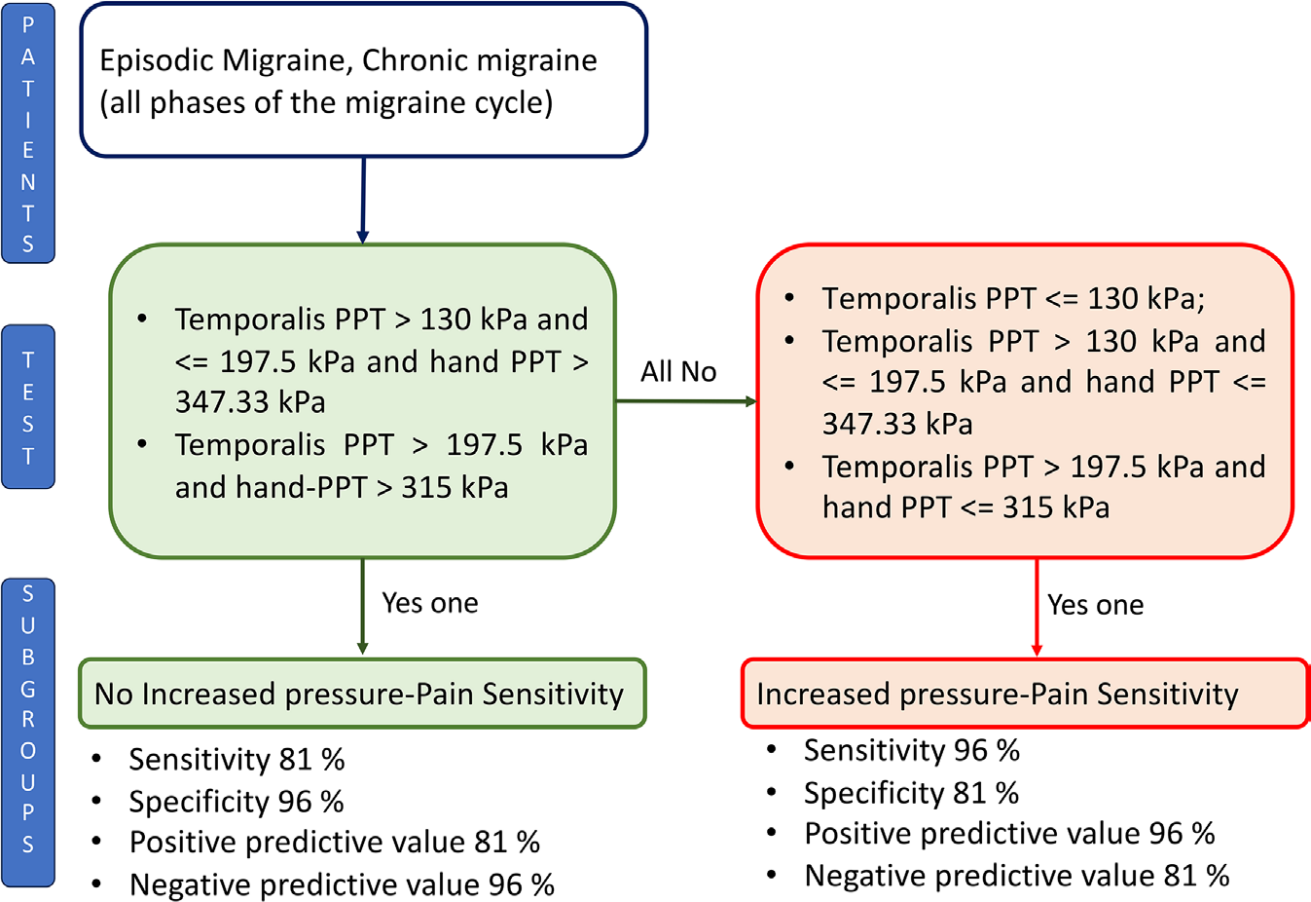
Sensitivity, specificity, positive predictive value, and negative predictive value of cut‐off values able to identify migraine patients with increased pressure pain sensitivity. PPT, pressure pain threshold.

A total of 161 (81%) migraine patients were included in the IPS group, while the remaining 37 (19%) were in the NoIPS group. The IPS group demonstrated deduced PPT values in all tested areas compared to the NoIPS group and a group of healthy subjects (Figure 1 and Table 1). Migraine patients were included in the IPS group if any of the following characteristics were present: (1) PPT over temporalis ≤ 130 kPa; (2) PPT over temporalis > 130 kPa and ≤ 197.5 kPa and PPT over the hand ≤ 347.33 kPa; (3) PPT over temporalis > 197.5 kPa and PPT over the hand ≤ 315 kPa. These three cut‐off values correctly included migraine patients in the IPS group with a sensitivity of 96%, a specificity of 81%, a positive predictive value of 96%, and a negative predictive value of 81% (Figure 2). The overall accuracy of the model was 93% (model error: mean = 0.07; Standard error = 0.02). The result of the cross‐validation analysis revealed an overall accuracy of 92% (model error: mean = 0.08; Standard error = 0.02).

**TABLE 1:** Difference in sex, age, BMI, and PPT across IPS migraine, NoIPS migraine and healthy controls.

	Controls (56)	NoIPS (37)	IPS (161)	Between group difference	Controls vs. NoIPS	Controls vs. IPS	NoIPS vs. IPS
Age, mean (SD)^a^	37.2 (14.3)	39.0 (13.0)	37.9 (11.7)	*ꭓ* ^2^ = 0.48, *p* = 0.785	*p* = 1.000	*p* = 1.000	*p* = 1.000
Sex, number (%)^b^				*ꭓ* ^2^ = 17.9, *p* < 0.001*	*p* = 0.435	*p* = 0.048*	*p* < 0.001*
Female	40 (71%)	21 (57%)	138 (86%)				
Male	16 (29%)	16 (43%)	23 (14%)				
BMI, mean (SD)^a^	22.1 (2.7)	23.1 (3.8)	23.1 (3.8)	*ꭓ* ^2^ = 2.42, *p* = 0.295	*p* = 0.759	*p* = 0.390	*p* = 1.000
PPT anterior column temporalis, mean kPa (SD)^c^	192.1 (75.5)	230.0 (52.5)	125.3 (48.3)	*F* = 45.9 *p* < 0.001*	*p* = 0.066	*p* < 0.001*	*p* < 0.001*
PPT middle column temporalis, mean kPa (SD)^c^	258.5 (113.9)	331.3 (90.9)	169.2 (67.5)	*F* = 49.4 *p* < 0.001*	*p* = 0.009*	*p* < 0.001*	*p* < 0.001*
PPT posterior column temporalis, mean kPa (SD)^c^	294.4 (127.1)	391.1 (126.7)	191.2 (73.1)	*F* = 51.4 *p* < 0.001*	*p* = 0.003*	*p* < 0.001*	*p* < 0.001*
PPT C1 left, mean kPa (SD)^c^	264.8 (138.9)	341.3 (98.7)	176.2 (63.4)	*F* = 40.8 *p* < 0.001*	*p* = 0.002*	*p* < 0.001*	*p* < 0.001*
PPT C1 right, mean kPa (SD)^c^	267.3 (129.6)	368.0 (111.9)	178.7 (71.4)	*F* = 47.0 *p* < 0.001*	*p* = 0.001*	*p* < 0.001*	*p* < 0.001*
PPT C2 left, mean kPa (SD)^c^	243.0 (105.3)	296.2 (112.1)	158.8 (62.1)	*F* = 44.2 *p* < 0.001*	*p* = 0.105	*p* < 0.001*	*p* < 0.001*
PPT C2 right, mean kPa (SD)^c^	246.9 (117.9)	297.4 (87.6)	162.1 (62.0)	*F* = 47.3 *p* < 0.001*	*p* = 0.060	*p* < 0.001*	*p* < 0.001*
PPT C4, left mean kPa (SD)^c^	249.1 (98.2)	286.7 (89.3)	158.9 (60.3)	*F* = 44.9 *p* < 0.001*	*p* = 0.327	*p* < 0.001*	*p* < 0.001*
PPT C4, right mean kPa (SD)^c^	251.0 (120.6)	305.3 (97.3)	168.6 (64.9)	*F* = 38.1 *p* < 0.001*	*p* = 0.080	*p* < 0.001*	*p* < 0.001*
PPT C6 left, mean kPa (SD)^c^	344.2 (150.4)	439.6 (183.4)	219.1 (99.2)	*F* = 40.2 *p* < 0.001*	*p* = 0.099	*p* < 0.001*	*p* < 0.001*
PPT C6 right, mean kPa (SD)^c^	366.1 (190.5)	470.5 (209.1)	221.6 (102.8)	*F* = 40.9 *p* < 0.001*	*p* = 0.083	*p* < 0.001*	*p* < 0.001*
PPT second MCPJ, mean kPa (SD)^c^	328.2 (135.9)	442.5 (126.5)	242.8 (82.7)	*F* = 38.6 *p* < 0.001*	*p* = 0.002*	*p* < 0.001*	*p* < 0.001*
PPT tibial anterior, mean kPa (SD)^c^	438.3 (211.4)	612.6 (177.4)	329.1 (140.8)	*F* = 32.3 *p* < 0.001*	*p* < 0.001*	*p* = 0.001*	*p* < 0.001*

Abbreviations: BMI, body mass index; IPS, increased pressure‐pain sensitivity; kPa, kilo pascal; MCPJ, metacarpophalangeal joint; n.s., non‐significant; NoIPS, no increased pressure‐pain sensitivity; PPT, pressure pain threshold; SD, standard deviation.

^a^ As data were not normally distributed, differences were investigated with the Kruskal‐Wallis test. The Mann–Whitney test was used to run post hoc analyses (Bonferroni corrected *p*‐value). Normality was assessed with the Shapiro–Wilk test.

^b^ Differences were investigated with the Chi‐square test and the Chi‐square test (2 × 2 contingency table) was used to run posthoc analyses (Bonferroni corrected *p*‐value).

^c^ ANCOVA controlling for age. The analysis was performed on log‐transformed data to fulfil the normality assumption. Normality was assessed with the Shapiro–Wilk test. Bonferroni‐adjusted posthoc analysis was performed to make single‐group comparisons.

* Significant at *p* < 0.05 (Bonferroni correction was performed in the posthoc analysis).

## 4 Discussion

The high internal validity suggests that the model could precisely predict the presence of IPS independently of the phase in which the assessment was done. These results suggest that the trigeminal and hand PPT cut‐off values presented in this study could be used in a clinical and research setting to identify patients with IPS, independently of the phase of the migraine cycle. In a clinical setting, identifying patients with IPS with simple bedside tests may be important, to design a tailored multidisciplinary management, targeting this aspect. Pressure‐pain hyperalgesia is often underestimated, and hence reliable tools are warranted. However, future longitudinal studies assessing the same patients across different migraine phases should be performed to confirm the validity of these cut‐off values.

The clinical validity of this assessment procedure should also be further investigated. Even if the presence of cutaneous allodynia is a promising biomarker to identify responders to acute and prophylactic treatments in migraine patients (Delussi et al. 2020; Ezzati et al. 2023; Hong et al. 2023; Pan et al. 2022; Pijpers et al. 2023), the ability of pressure‐pain hyperalgesia to predict treatment response has been less studied and is still debatable (Börner et al. 2022; Peng et al. 2022; Schwarz et al. 2021). Cutaneous allodynia and pressure‐pain hyperalgesia could be considered two different proxies of the same complex phenomenon (increased sensitization mechanisms), and their results do not always correlate (Di Antonio, Castaldo, et al. 2022; Di Antonio et al. 2024). Thus, it could not be speculated that the promising role of allodynia as a biomarker to predict treatment response could also apply to pressure‐pain hyperalgesia and future studies that specifically assess the role of pressure‐pain hyperalgesia in predicting treatment response are needed.

## Conflicts of Interest

The authors declare no conflicts of interest.
